# Music and Video Gaming during Breaks: Influence on Habitual versus Goal-Directed Decision Making

**DOI:** 10.1371/journal.pone.0150165

**Published:** 2016-03-16

**Authors:** Shuyan Liu, Daniel J. Schad, Maxim S. Kuschpel, Michael A. Rapp, Andreas Heinz

**Affiliations:** 1 Department of Psychiatry and Psychotherapy, Charité Campus Mitte, Charité Universitätsmedizin Berlin, Berlin, Germany; 2 Social and Preventive Medicine, Universität Potsdam, Potsdam, Germany; University of Akron, UNITED STATES

## Abstract

Different systems for habitual versus goal-directed control are thought to underlie human decision-making. Working memory is known to shape these decision-making systems and their interplay, and is known to support goal-directed decision making even under stress. Here, we investigated if and how decision systems are differentially influenced by breaks filled with diverse everyday life activities known to modulate working memory performance. We used a within-subject design where young adults listened to music and played a video game during breaks interleaved with trials of a sequential two-step Markov decision task, designed to assess habitual as well as goal-directed decision making. Based on a neurocomputational model of task performance, we observed that for individuals with a rather limited working memory capacity video gaming as compared to music reduced reliance on the goal-directed decision-making system, while a rather large working memory capacity prevented such a decline. Our findings suggest differential effects of everyday activities on key decision-making processes.

## Introduction

Decision making is an integral part of everyday life. People have to make choices within diverse decision-making environments. It has been found that taking a break benefits cognitive performance [1–5], possibly by providing a favorable condition for offline replay by minimizing the incoming interference information [6]. However, the effects of taking a break during the decision-making process remain to date to be explored.

Decision making is influenced by two systems: the habitual and the goal-directed system. These two systems can also be described computationally as model-free versus model-based, or by the terms retrospective versus prospective [7]. We recently observed significant correlations between goal-directed and model-based decision making, supporting the notion that these concepts address similar behavioral decision-making systems [8]. When we rely on the habitual (model-free) system, we simply repeat the actions that lead to a gain in the past. When using the goal-directed (model-based) system, choices are guided by the likelihood of affective outcomes that are predicted by a model of the environment [7]. Computational models have described habitual behavior as depending on model-free retrospective reinforcement, and goal-directed behavior to rely on model-based prospective planning [9, 10]. Adaptive behavior depends on the ability to flexibly regulate the respective contributions of the habitual and goal-directed systems [10].

A number of factors have been discovered to interact with and shape these two systems, with cognitive abilities such as working memory found to be one of the core factors [7, 9, 11–13]. Indeed, dual-task conditions [14], electromagnetic stimulation (TMS) to the dorsolateral prefrontal cortext (dlPFC) [15], as well as acute [14] or chronic [16] stress are thought to impair executive resources underlying working memory and were found to impair goal-directed decision-making, inducing a relative shift towards habitual behavioral control. Experimental manipulations are often found to interact with baseline working memory capacity, such that (a) goal-directed reasoning is particularly impaired in individuals with a rather limited working memory capacity, and (b) individual differences in baseline working memory capacity predict reliance on goal-directed decision-making under conditions where working memory is impaired [14, 15].

Other cognitive abilities are also found to moderate the balance between goal-directed versus habitual choice systems. Importantly, goal-directed control increases with higher levels of processing speed, particularly in individuals with a large working memory capacity [13]. Moreover, an inverted U-shaped curve was found for the effect of processing speed on habitual choice, indicating that habitual choice is strongest at medium levels of processing speed [13]. Likewise, executive resources and verbal knowledge [17] seem to play important roles in reward-based decision making.

We hypothesized that other potential factors, such as different activities during a break, might shape the balance between habitual and goal-directed decision-making systems. We chose two common break activities: listening to music and playing a video game. Music can act as a catalyst for cognitive abilities: it has been demonstrated that musical education can enhance working memory [18, 19]. Rauscher found that listening to a Mozart sonata increases subsequent spatial reasoning ability [20]. However, the effect may rather depend upon mood, arousal, or enjoyment [21] and little is known about the specific aspects of music that contribute to the transfer effects on learning.

Playing video games can result in a wide range of behavioral benefits, including enhancement of task performance, spatial cognition, processing speed, task switching and level of reasoning in decision making [22]. At the same time, video games have been associated with a variety of negative outcomes. For example, the sound of a video game could disturb the players’ concentration on learning [22, 23] and induce physiological stress while gaming [24]. So far, research has rarely explored the effects of video games interleaved with different types of learning, though a few studies have already found an effect on memory performance [1, 25]. Recently, we [26] found that playing video games reduces subsequent working memory performance over time, likely because gaming during breaks prevents recovery of depleted executive resources, eliciting failures in sustained attention and increasing “mind wandering”.

To date, effects of different break activities on a decision-making task have not been investigated. Given their known influence on working memory, we predicted that the type of activity engaged in during breaks might significantly affect decision-making systems and shape subsequent performance. Specifically, we assumed that listening to music might enhance goal-directed decision making. Based on our previous finding that video gaming impairs working memory [26], we expected gaming to reduce goal-directed decision making. Moreover, we aimed to explore whether this effect is particularly prominent in individuals with a low working memory capacity, suggesting an influence of baseline working memory capacity on individual differences in goal-directed control. We used a within-subjects design to have subjects perform a sequential two-step Markov decision task [10, 27], which involves decision preferences that change on a trial-by-trial basis to access these two decision-making systems.

## Methods

### Subjects

Thirty three right-handed healthy native German subjects (17 female; age range: 19–32, *Mean* = 24.6, *SD* = 3.5) were recruited in Berlin. Subjects were screened for major psychiatric disorders (SCID-I screening questionnaire). Basic information was gathered: social and demographical data, music listening habits (time spent on listening to music per week in the past 12 months, types of music listened to) and video gaming experience (time spent on playing games per week in the past 12 months, types of games played). Subjects were given detailed information and provided fully informed written consent. The study was approved by the Ethics Committee of the Charité –Universitätsmedizin Berlin and was performed in accordance with the ethical standards laid down in the 1964 Declaration of Helsinki. The target sample size was estimated on the basis of our previous study on cognitive abilities in decision-making [13]. No subjects’ data was excluded from analysis.

### Neuropsychological battery

Subjects underwent neuropsychological testing including verbal knowledge [28], fluid intelligence cognitive speed [29] and memory and executive functioning [29–32] (Table 1).

**Table 1:** Socio-demographic information and results from a neuropsychological battery for the 33 healthy subjects who participated in experiment.

	N = 33
Age (years)	24.64 (0.61 ^a^)
Education (years)	16.29 (0.44)
Fluid Intelligence Cognitive Speed (DSST, numbers)	86.67 (1.67)
Verbal Knowledge (MWT-B, numbers)	27.36 (0.63)
Verbal Memory (wordlist, numbers)	9.12 (0.19)
Verbal Working Memory (DS, numbers)	7.39 (0.31)
Semantic Verbal Fluency (SVF, numbers)	29.42 (1.04)
Executive Functioning (TMT-A, seconds)	27.61 (1.52)
Executive Functioning (TMT-B, seconds)	55.63 (3.15)

^a^Standard error of the mean (SEM).

Note: Cognitive Speed was assessed by the Digit Symbol Substitution Test (DSST) from the WAIS-R [29]; Verbal IQ was assessed by the German Vocabulary Test (Mehrfachwahl-Wortschatz-Intelligenztest, MWT-B [28]); Verbal memory was assessed by Wordlist from the Consortium to Establish a Registry for Alzheimer's Disease (CERAD [30]); Verbal Working Memory was assessed by the Digit Span (DS) Backwards Test [29]; One-minute Semantic Verbal Fluency (SVF) was tested for the category “animals” (Verbale Flüssigkeit Tiere [31]); Executive Functioning was assessed by the Trail Making Test (TMT) A and B [32].

### Break activity scenarios

To evaluate the effects of different break activities on the habitual and goal-directed decision-making systems, subjects were instructed to engage three times (i.e. once before and twice during the main task) in “listening to music” or “playing a video game”, for 8:30 min per break. For the “listening to music” condition, subjects were instructed to listen to Mozarts “Sonata for Two Pianos in D Major, KV. 448—Allegro con spirito” over headphones; for the “playing a video game” condition, subjects were instructed to play the “Angry Birds” video game (www.angrybirds.com) on a laptop computer. We chose Mozarts Sonata piece because it has been a major musical piece in empirical investigations of the effects of music on cognitive functions and has been used in a large range of studies [20, 21, 33]. We chose the popular mobile game Angry Birds game because playing Angry Birds utilizes concepts of qualitative spatial representation, utility function and decision making under uncertainty [33, 34].

### Procedure

**Fig 1:**
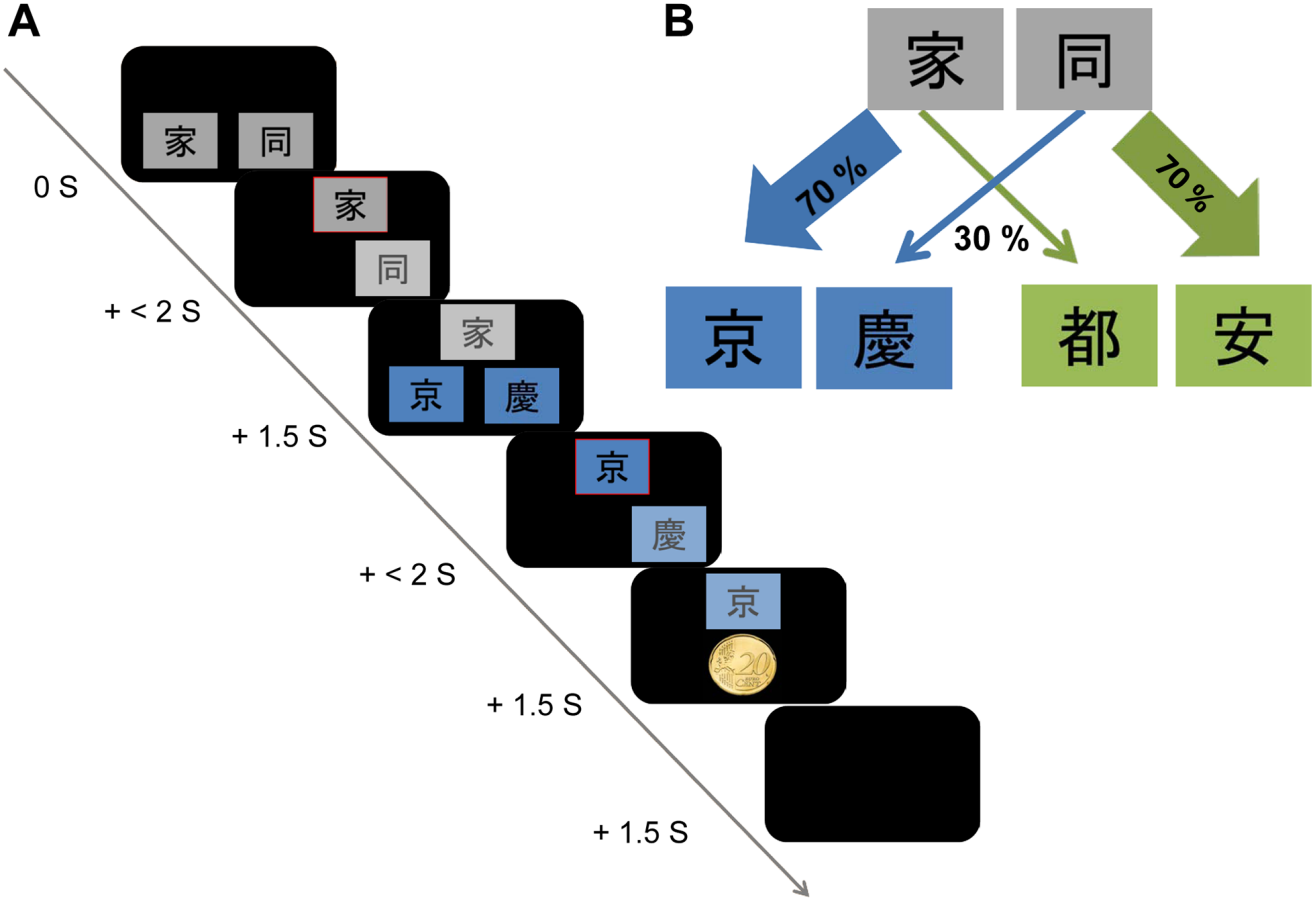
Two-step decision task. (A) Trial structure. Each trial consisted of choices at two steps. Step 1 involved the first choice between two abstract gray stimuli (Chinese characters, not known to German subjects). The chosen stimulus was framed with red color in the center-top of the screen for 1.5s. Subsequently, subjects were presented with another stimulus pair in step 2. The second choice was rewarded with money (20 cents) or nothing. (**B**) The transitions from step 1 to step 2 remained fixed, with 70% and 30% of all trials as respectively common and rare transitions. The reward probabilities for each stimulus in step 2 changed independently between 25% and 75%, based on Gaussian random walks with reflecting boundaries [10]. Win probabilities varied, therefore, as a function of the trial number.

The sequential two-step Markov decision task drew on on Wunderlich, Smittenaar, & Dolan [27] to assess the relative degree of habitual vs. goal-directed decision making when this decision making is preceded and interrupted by breaks of either listening to music or playing a video game. The version of the task was identical to on Wunderlich, Smittenaar, & Dolan [27] except for different stimulus images (see Fig 1). The task consisted of two subsequent steps, each demanding a choice between two stimuli. During the first step (step 1), two stimuli were presented and subjects were requested to choose one. This choice probabilistically determined (i.e. a common (70%) or a rare (30%) transition) which pair of stimuli was presented at the second step. During the second step (step 2), subjects were presented with another pair of stimuli and again requested to choose one. This second choice was either rewarded with money (20 cents) or nothing. The reward signal during the task was the amount of 20 cents for each trial. To enhance subjects’ motivation, subjects were also advised that the top three performers could also expect to be rewarded with an unspecified, but worthy and pleasant surprise at the end of the experiment. The surprise was valuable gift boxes of chocolates.

Prior to the experiment, subjects were given very detailed information about the structure of the task; they were informed that the reward probabilities in step 2 would change (and were shown exemplary sample random walks), but those controlling the transitions from step 1 to step 2 would remain fixed and be primarily associated with one or the other of the second-step states.

Subjects underwent 50 practice trials with a separate set of stimuli before starting the main task. Immediately after practice, subjects engaged in an 8:30 min break of either gaming or listening to music. Subsequently, they started the main task, which consisted of 201 trials with two breaks (of listening to music or video gaming) after trial 67 and 134. A repeated measures design with within-subjects factors of two break activities (music vs. gaming) and two versions of two-step task was applied and the order was counterbalanced across subjects.

Immediately after every task performance, we asked subjects to rate the task difficulty and their ability to concentrate on the task, as well as their enjoyment of the break activity and the extent to which they thought about the task during the break by using visual analogue scales (VAS) [35].

### Analysis

We used the *stats* package for data analysis and *ggplot2* package (Wickham, Springer New York, USA) for graphics in the *R system for statistical computing (version 3*.*1*.*0*; www.r-project.org).

Statistical tests of the VAS questionnaires were performed using SPSS Statistics Version 18 (SPSS Inc., Chicago, IL, USA). Differences were considered significant at *p* < .05 and highly significant at *p* < .001. We used paired *t*-test for pairwise comparisons of the respective game and music conditions (two-tailed *p* values were assumed).

### Computational modelling

We used the computational dual-control model by Daw and co-workers [10] (for a detailed description of our implementation, see Schad and co-workers [13]) and adapted it to analyse the observed data: following Otto and co-workers [14], we parameterized the model to assess separate weights for the habitual versus the goal-directed system (instead of assessing their relative balance as in [10, 13]). The model suggests reward-based choice to originate from two distinct systems of model-free versus model-based reinforcement learning (RL), thought to reflect habitual versus goal-directed learning. Model-free RL is implemented as SARSA (*λ*) temporal difference learning [36] and model-based RL implements Bellman’s equation [37], assuming that expected maximal outcomes at the second stage are rationally weighted by their (transition) probabilities, which are taken to be known and fixed (cf. Daw and co-workers [10]). Throughout the task, both algorithms thus learn expected (Q-) action values.

Both algorithms operate in a state space with a single starting state at first-stage. From this first-stage state, two possible actions induce random transitions (with fixed probabilities .3/.7) to one out of two second-stage states. Both second-stage states each allow two possible actions, which deterministically lead to one of four distinct final states, where no further actions are possible, but reward outcomes are delivered. Win-trials are coded via presentation of a reward value of one, and in no-win trials a reward of zero is obtained. For choice, action (Q-) values from both systems are combined according to each system’s estimated weight, and fed through a softmax-function to obtain action probabilities. For details on the original model see Daw and co-workers [10] and Schad and co-workers [13].

The model contains seven free parameters: (*i*+*ii*) *α*, learning rate for the first (*α*_*1*_) and the second (*α*_*2*_) step; (*iii*) *λ*, the reinforcement eligibility parameter, determines the relative degree of second-step reward prediction errors to update first-step model-free (habitual) values; (*iv*) *β*_*2*_ the inverse temperature parameter controls how deterministic choices are at the second step; (*v*+*vi*) separate weights for the model-free and the model-based system reflect contributions of habitual (*β*_*HB*_) versus goal-directed (*β*_*GD*_) decision-systems to choice; (*vii*) *p*, first-step choice perseveration or stickiness.

In the present study, subjects performed the two-step task twice and breaks filled with either music or gaming were interleaved with each task performance. In the computational modeling, we estimated the parameter values of each subject for each of these two conditions separately. For bounded parameter estimation and due to the normal distribution assumption in statistical testing, we transformed bounded model parameters to an unbounded scale via a logistic transformation [x’ = log(x/(1-x))] for parameters *α* and *λ*, and via an exponential transformation [x’ = exp(x)] for parameters *β*.

We estimated individual model parameters via a Bayesian fitting procedure. Based on a rather uninformative broad prior distribution (uncorrelated normal distributions; *M* = 0; *SD* = 100), we obtained maximum a posteriori (MAP) estimates for each individual subject and each session of two-step performance, assuming the same prior for gaming and music. For estimating individual MAP parameters, we performed unconstrained iterative optimization using the *fminunc* function in Matlab. Repeated estimation runs with random starting values (normally distributed; *M* = 0; *SD* = 0.1) yielded similar group-level results, such that significant and marginal results were either significant or marginal in each of six independent estimation runs. In an additional confirmatory analysis, we estimated the (uncorrelated) prior distribution using one step of Expectation Maximization (EM) [12, 13], treating two sessions of 2-step task performance per subject as independent. Individual MAP estimates based on this prior yielded a pattern of non-/significant results that was similar to the one reported below for the described Bayesian analysis.

### Statistical testing

We used linear mixed-effects models as implemented in the *lme4* package [38] in the R system for statistical computing (www.r-project.org) in order to regress individual MAP parameter estimates on the fixed effects predictor break condition (music versus gaming) and on random subject intercepts. We interpreted differences in model parameters between experimental conditions as evidence that the experimental manipulation affected decision-making during task performance in a way that can be understood as a change in this specific decision parameter (for a similar approach see e.g., Otto and co-workers [14, 39]; Smittenaar and co-workers [15]; Schad and co-workers [13]). As an alternative possibility, experimental conditions may change the structure of the decision-process, that is, the computational model generating the data for each subject [10, 40], which should be tested in future research.

In a second and more explorative analysis step we tested the hypothesis based on previous research [14, 15, 26] that break effects may impair model-based control in individuals with a low baseline working memory capacity, but that a high working memory capacity may protect individuals from gaming-related decline [14]. To this end, we added the fixed-effect interaction between break activity (music versus gaming) and Digit Span as a fixed-effect. Digit Span scores were z-transformed prior to analysis to yield standardized regression coefficients. To assess break-effects for individuals with a large versus small working memory capacity, we used the standard regression technique [41] of re-centering the (z-standardized) Digit Span score variable (as an indicator of working memory capacity) to values of one standard deviation above or below its mean for this analysis. Moreover, as certain cognitive abilities are also known to influence the goal-directed decision-system [13], we controlled for this influence by adding measures for working memory (Digit Span), processing speed (DSST and TMT-A), and verbal knowledge (MWT) as control variables to the fixed-effects predictors.

Subsequently, we performed exploratory tests for the corresponding interactions of break effects with the other cognitive ability scores. Finally, we tested the effect of break activity (music versus gaming) on the other model parameters, Bonferroni-correcting for the multiple (i.e., six) explorative statistical tests.

Throughout the analyses, we investigated directed hypotheses using one-tailed testing.

Previous evidence suggests that factors like stress [14] or electromagnetic stimulation (TMS) to the dlPFC [15] reduce goal-directed control only in individuals with low working memory, and thus enhance an influence of individual differences in working memory on goal-directed control. Accordingly, we here expected that playing computer games during breaks (but not listening to music) may may impair goal-directed control only among individuals with a low working memory capacity, such that individual differences in working memory would influence the weight of the goal-directed decision-system after gaming.

## Results

Social demographic information and a neuropsychological battery of the sample are presented in Table 1. Our data on music listening habits and video gaming experience showed that subjects spent on average 10.5 hours per week on listening to music, and 3.4 hours on gaming, *t*(32) = -4.36, *p* < .001. There were four frequent gamers according to the definition by [42] and no professional musicians among the subjects. There was no significant difference in the VAS ratings of the difficulty of two task versions, *t*(32) = .46, *p* = .651, subjects’ ability to concentrate on the task after gaming and listening to music, *t*(32) = .27, *p* = .793, and the extent to which they thought about the task during the breaks, *t*(31) = -.56, *p* = .581. Subjects reported that on average they enjoyed gaming 21% more than listening to music, *t*(31) = 3.89, *p* < .001.

**Fig 2:**
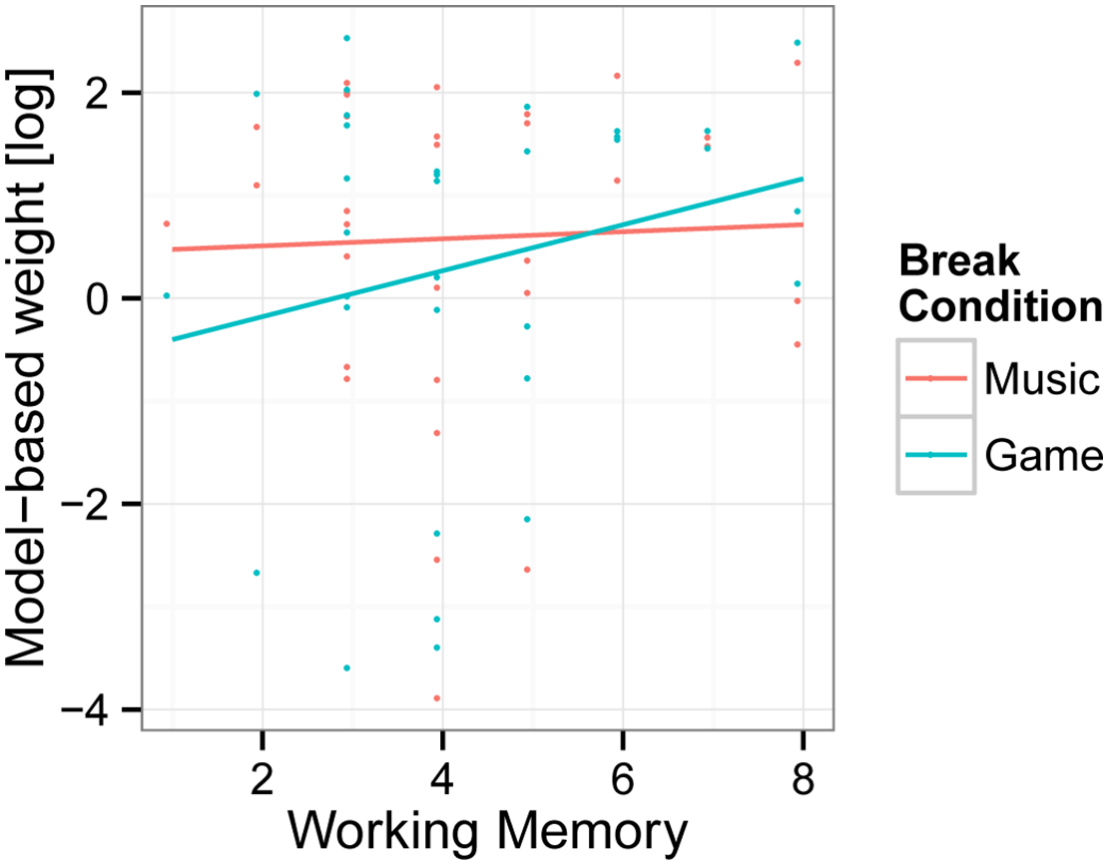
Computational model parameter estimates. The model parameter *β*_*GD*_, estimating the weight of the goal-directed systems to behavioral control, is displayed as a function of working memory capacity (Digit Span score) for the break conditions gaming (green points and regression lines) versus music (red).

We estimated individual maximum a posteriori (MAP) model parameters and computed summary statistics for these estimates, which are presented in Table 2. The plot of estimated differences in model parameter as a function of working memory capacity (Digit Span score) between the break conditions gaming versus music are shown in Fig 2.

**Table 2:** Computational model parameters: maximum a posteriori (MAP) estimates (bounded model-scale).

	*α*_*1*_	*α*_*2*_	*β*_*HB*_	*β*_*GD*_	*λ*	*β*_*2*_	*ρ*
Mean	0.60	0.67	2.42	3.33	0.79	3.13	0.76
25%	0.31	0.58	0.91	0.84	0.56	1.48	0.36
75%	0.93	0.88	3.22	5.19	0.98	4.18	1.11

First, we tested whether gaming reduces the *ω* parameter overall, but found no significant effect (*p* = .14). Next, we tested the hypothesis that gaming would impair goal-directed decision-making in individuals with a small working memory, and that a large working memory would prevent such decline decline [14, 26]. Consistent with this hypothesis, we found that gaming compared to music reduced the *β*_*GD*_ parameter in individuals with a small Digit Span score (*b* = -0.41, *SE* = 0.22, *df* = 31, *t* = -1.87, *p* = .04), but did not affect goal-directed control in individuals with a large Digit Span score (*p* = .76; for the interaction: *b* = 0.24, *SE* = 0.16, *df* = 31, *t* = 1.53, *p* = .07). Likewise, we investigated the hypothesis that a small working memory capacity would be associated with reduced model-based control after gaming, but not after music [15, 26]. Consistent with this expectation, we found that an increase of the model-based weight (*β*_*GD*_) with Digit Span score was not significant overall (*b* = 0.23, *p* = 0.16) or after music (*b* = 0.11, *p* = .33), but was marginally reliable after gaming (*b* = 0.35, *SE* = 0.24, *df* = 35, *t* = 1.45, *p* = .08).

Explorative analyses further suggested that the *β*_*GD*_-parameter was increased in individuals with a high DSST score (*b* = 0.66, *SE* = 0.22, *df* = 28, *t* = 3.0, *p* = .003), replicating previous findings that high processing speed enhances goal-directed decision-making [13]. The influence of our second measure of processing speed (TMT-A: *p* = .18) and of verbal knowledge (MWT, *p* = .26) were not significant in the present sample. We also explored whether the break effect (difference between gaming and music) interacted with one of these other cognitive abilities, but found no significant effect (*p*-values > .30).

Last, we explored whether break activities influenced one of the other six model parameters. We found only one significant effect of break activity on the repetition parameter (*b* = -0.16, *SE* = 0.07, *df* = 32, *t* = -2.3, *p* = .03), indicating that gaming reduced choice stickiness, but this effect did not survive Bonferroni correction for multiple (i.e., six) comparisons (*p* = .16).

## Discussion

We studied how habitual and goal-directed decision-making systems are affected by different break activities (i.e., listening to music and playing a video game). We found that gaming reduced reliance on the goal-directed system in individuals with a rather low working memory capacity, while leaving the habitual system intact. These results were consistent with our expectation that playing video games during break periods may impair working memory resources and thus interfere with goal-directed planing.

### Gaming reduced goal-directed decision making in low working memory individuals

We recently observed [26] that video gaming compared to listening to music reduced working memory performance in the *n*-back task over time. In line with these previous findings, we have found that gaming reduces reliance on the goal-directed decision system in low working-memory individuals, suggesting that gaming may interfer with working memory resources needed for goal-directed planning. This finding is congruent with previous research showing that taxing executive resources can impair goal-directed choice particularly in individuals with low baseline working memory capacity, but often has little effect in high working-memory individuals [14, 15]. Gaming may thus have rather subtle interfering effects on executive functioning: during decision-making, individuals with high levels of executive resources appear to be able to compensate for these effects and maintain high levels of goal-directed control. Individuals whose executive resources are limited, however, may not be able to guard against such negative influences and impaired executive resources may induce a decline in goal-directed planning.

A recent computational proposal [11] frames the arbitration between habitual vs. goal-directed decision making as a tradeoff between time cost and behavioral flexibility, both of which are high in goal-directed and low in habitual decision making. Gaming might create internal time pressure and heavily tax attention which may lead low-working memory subjects to reduce cognitive and time costs by immediately scaling down goal-directed choice and elevating habitual choice at the initial step.

How may such an influence of gaming on working memory processes in goal-directed control be moderated by additional factors? Interestingly, in our previous study [26], the decremental influence of gaming on working memory was associated with mind wandering and impaired concentration, suggesting that these factors may contribute to the present findings. As an additional possible explanation, gaming may induce stress [24], which could in turn decrease cognitive performance [22]. The sound of a video game could disturb the players’ concentration on learning [22, 23] and induce physiological stress while gaming [24]. Such stress may tax cognitive resources and consequently inhibit more sophisticated ways of goal-directed decision making but spare more parsimonious habitual decision making [14, 43]. Thus subjects might not integrate the information of each step into a cohesive model in order to optimize choice selections. An interference of stress with prefrontal-dependent functions [44] may thus contribute to the negative effects on goal-directed choice.

Recent work suggests that goal-directed processes impose considerable demands on central executive resources [9, 11], which rely on the prefrontal cortex [45]. These central executive resources including processing speed and working memory capacity may interact to moderate the tradeoffs between habitual and goal-directed systems [13], and may thus provide particular vulnerabilities to interference with executive functions.

### Limitations

The above effects of breaks on the sequential two-step Markov decision task have been observed in young, well-educated subjects. Other subject groups and other types of music or games, e.g. self-selected [46], may result in different findings. Our sample did not allow for a valid comparison of gamers and non-gamers, musicians and non-musicians and the small sample size limits generalizability and requires independent replications. Our limitations of our study are potential transfer effects, i.e. that listening to music or playing video games may affect performance over a longer period of time. We did not measure physiology responses (e.g. heart rate and skin conductance). This limits our ability to interpret findings that might have resulted from subjects’ arousal level during breaks and decision making.

## Conclusions

We examined whether typical everyday activities that people engage in during a break (i.e. listening to music and playing a video game) interact with decision making. We did not find support for our hypothesis that gaming reduces overall goal-directed decision-making. However, we found that break effects depended on baseline working memory: in individuals with a rather low working memory capacity video gaming induced a relative shift from goal-directed to habitual decision making as compared to listening to (Mozart’s) music. A rather high working memory capacity, on the other hand, prevented such a decline in goal-directed control, suggesting that (as compared to music) gaming may prevent recovery of executive resources during breaks, leading to a decline in resources available for goal-directed planning. Understanding learning mechanisms during break activities may help to develop better procedures for rest and recuperation and help guide further research into the effects of video gaming on young adults, specifically breaks in between learning sessions. Also, our research can help to develop recommendations for parents, who have to decide whether they should ban their kids from playing video games during learning or whether they can tolerate or even encourage them to play.
